# Polytrauma Is Associated with Increased Three- and Six-Month Disability after Traumatic Brain Injury: A TRACK-TBI Pilot Study

**DOI:** 10.1089/neur.2020.0004

**Published:** 2020-07-23

**Authors:** John K. Yue, Gabriela G. Satris, Cecilia L. Dalle Ore, J. Russell Huie, Hansen Deng, Ethan A. Winkler, Young M. Lee, Mary J. Vassar, Sabrina R. Taylor, David M. Schnyer, Hester F. Lingsma, Ava M. Puccio, Esther L. Yuh, Pratik Mukherjee, Alex B. Valadka, Adam R. Ferguson, Amy J. Markowitz, David O. Okonkwo, Geoffrey T. Manley

**Affiliations:** ^1^ Department of Neurological Surgery, University of California San Francisco, San Francisco, California, USA.; ^2^ Brain and Spinal Injury Center, Zuckerberg San Francisco General Hospital, San Francisco, California, USA.; ^3^ Department of Neurological Surgery, University of Pittsburgh Medical Center, Pittsburgh, Pennsylvania, USA.; ^4^ Department of Psychology, University of Texas, Austin, Texas, USA.; ^5^ Department of Public Health, Erasmus Medical Center, Rotterdam, The Netherlands.; ^6^ Department of Radiology, University of California San Francisco, San Francisco, California, USA.; ^7^ Department of Neurological Surgery, Virginia Commonwealth University, Richmond, Virginia, USA.

**Keywords:** disability, functional outcome, outcome measure, polytrauma, traumatic brain injury

## Abstract

Polytrauma and traumatic brain injury (TBI) frequently co-occur and outcomes are routinely measured by the Glasgow Outcome Scale-Extended (GOSE). Polytrauma may confound GOSE measurement of TBI-specific outcomes. Adult patients with TBI from the prospective Transforming Research and Clinical Knowledge in Traumatic Brain Injury Pilot (TRACK-TBI Pilot) study had presented to a Level 1 trauma center after injury, received head computed tomography (CT) within 24 h, and completed the GOSE at 3 months and 6 months post-injury. Polytrauma was defined as an Abbreviated Injury Score (AIS) ≥3 in any extracranial region.

Univariate regressions were performed using known GOSE clinical cutoffs. Multi-variable regressions were performed for the 3- and 6-month GOSE, controlling for known demographic and injury predictors. Of 361 subjects (age 44.9 ± 18.9 years, 69.8% male), 69 (19.1%) suffered polytrauma. By Glasgow Coma Scale (GCS) assessment, 80.1% had mild, 5.8% moderate, and 14.1% severe TBI. On univariate logistic regression, polytrauma was associated with increased odds of moderate disability or worse (GOSE ≤6; 3 month odds ratio [OR] = 2.57 [95% confidence interval (CI): 1.50-4.41; 6 month OR = 1.70 [95% CI: 1.01-2.88]) and death/severe disability (GOSE ≤4; 3 month OR = 3.80 [95% CI: 2.03-7.11]; 6 month OR = 3.33 [95% CI: 1.71-6.46]). Compared with patients with isolated TBI, more polytrauma patients experienced a decline in GOSE from 3 to 6 months (37.7 vs. 24.7%), and fewer improved (11.6 vs. 22.6%). Polytrauma was associated with greater univariate ordinal odds for poorer GOSE (3 month OR = 2.79 [95% CI: 1.73-4.49]; 6 month OR = 1.73 [95% CI: 1.07-2.79]), which was conserved on multi-variable ordinal regression (3 month OR = 3.05 [95% CI: 1.76-5.26]; 6 month OR = 2.04 [95% CI: 1.18-3.42]). Patients with TBI with polytrauma are at greater risk for 3- and 6-month disability compared with those with isolated TBI. Methodological improvements in assessing TBI-specific disability, versus disability attributable to all systemic injuries, will generate better TBI outcomes assessment tools.

## Introduction

Traumatic brain injury (TBI) remains a significant cause of injury-related morbidity and mortality, with an annual incidence of more than 2.5 million cases in the United States and more than 50 million cases worldwide.^1,2^ Multi-system trauma is common in the setting of TBI, with reported incidences of up to 70% across large population-based cohorts.^3,4^ In the trauma literature concurrent TBI is linked to poorer prognoses;^5,6^ however, the impact of polytrauma on TBI outcomes remains understudied, especially in the civilian population. Proper outcome assessment tools are critical for capturing divergence in outcomes attributable to polytrauma after TBI. However, there are no validated tools for evaluating the impact of extracranial injuries on TBI outcomes.

For nearly 4 decades, the Glasgow Outcome Scale-Extended (GOSE) has remained the measure of choice for standard outcome assessment after TBI.^7–9^ The GOSE is a global disability measure that does not differentiate between disability from injury to the brain versus disability from extracranial injuries. Nevertheless, the GOSE has been the principal measure accepted by the U.S. Food and Drug Administration for primary outcomes^10^ and overall functional outcomes in TBI clinical trials.^8^

We hypothesized that peripheral injuries from polytrauma may alter and/or confound TBI outcomes as assessed using the GOSE. To pursue this question, we utilized prospective data from three U.S. Level 1 trauma centers to investigate the associations between polytrauma and 3- and 6-month GOSE. We present univariate and multi-variable outcomes associated with polytrauma versus isolated TBI, and discuss implications and solutions on TBI outcome assessment in the setting of polytrauma.

## Methods

The prospective, multi-center Transforming Research and Clinical Knowledge in Traumatic Brain Injury Pilot (TRACK-TBI Pilot) study (ClinicalTrials.gov Registration: NCT01565551) was conducted at three U.S. Level 1 trauma centers (the University of California San Francisco - Zuckerberg San Francisco General Hospital [San Francisco, California], the University of Pittsburgh Medical Center [Pittsburgh, Pennsylvania], and the University Medical Center Brackenridge [Austin, Texas]) using the National Institute of Neurological Disorders and Stroke (NINDS) TBI Common Data Elements (CDEs).^11^ Inclusion criteria for the TRACK-TBI Pilot were acute external force trauma to the head and presentation to a participating center, and a clinically indicated head computed tomography (CT) scan within 24 h of injury. Exclusion criteria were pregnancy, ongoing life-threatening disease (e.g., end-stage malignancy), police custody, involuntary psychiatric hold, and non-English speakers due to multiple outcome measures administered and/or normed only in English.

Eligible subjects were enrolled by convenience sampling from the years 2010 to 2012. Institutional Review Board (IRB) approval was obtained at each participating site, and the overall study received approval from the IRB of record at the University of California San Francisco (study #10-00111). Informed consent was obtained from each subject, or proxy, prior to enrollment. Subjects enrolled by surrogate consent were re-consented, if cognitively able, during the course of clinical care and/or follow-up time-points for study participation.

**FIG. 1.**
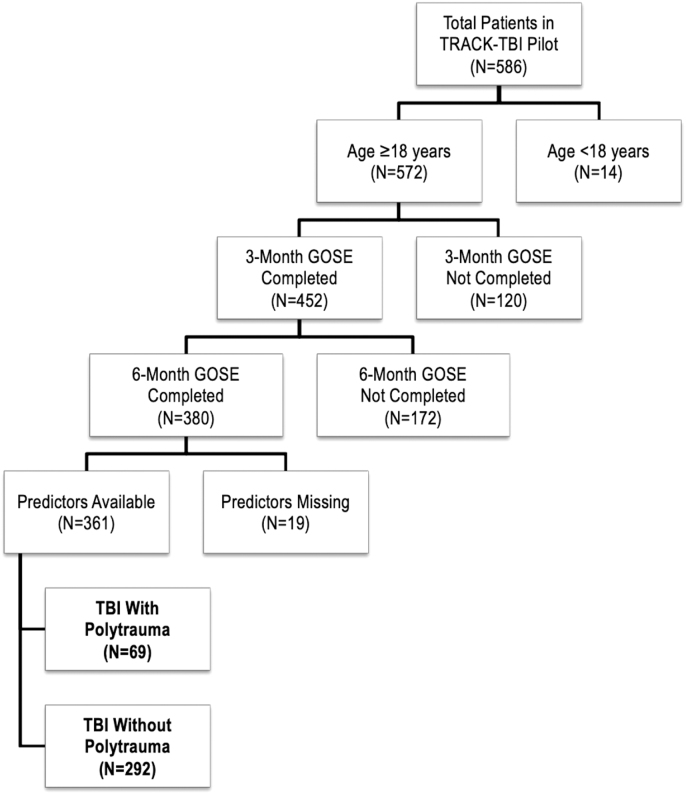
Flowchart of included patients.

The goal of the current analysis was to evaluate associations between multi-system trauma versus isolated TBI with baseline characteristics and outcome. Therefore, all subjects ≥18 years of age who completed the 3- and 6-month GOSE were included. Multi-system trauma was the variable of interest, defined as an Abbreviated Injury Score (AIS) of ≥3 in any extracranial body system; the AIS has been utilized in prior trauma and TBI literature.^12–16^ The flowchart of included subjects is shown in Figure 1.

### Demographic and clinical variables

Subjects were assessed by in-person interview and medical record review for demographics and baseline medical history, as well as clinical and injury history variables upon emergency department admission in accordance with the NINDS CDEs version 1.^17^ If admitted to the hospital, subjects were followed for the entirety of their hospital course.

### Neuroimaging

All subjects received a head CT within 24 h of injury as part of their clinical evaluation for TBI. Head CTs were read and coded by a central board-certified neuroradiologist blinded to subject characteristics in accordance with the NINDS CDEs version 1 for neuroimaging.^18^ The Marshall CT classification score^19,20^ was utilized to control for increasing TBI severity beyond presence/absence of intracranial lesions.

### Three- and six-month outcomes

The GOSE provides an overall measure of disability based on consciousness, independence inside and outside the home, employment/employability, social/community participation, and presence of post-concussion symptomatology.^7^ Scores are: 1 = dead, 2 = vegetative state, 3 = lower severe disability (e.g., able to carry out activities of daily living [ADLs] independently for no more than 8 h per day), 4 = upper severe disability (e.g., able to carry out ADLs for >8 h per day, able to make financial decisions and travel locally), 5 = lower moderate disability (non-competitive work or inability to work and/or inability to return to social activities and/or constant psychological disturbance), 6 = upper moderate disability (reduced work capacity and/or >50% reduced social participation and/or weekly psychological disturbance), 7 = lower good recovery (post-concussion symptoms and/or <50% reduced social participation and/or occasional psychological disruption), and 8 = upper good recovery (recovery back to pre-injury status without notable new deficits). In the TRACK-TBI Pilot, the GOSE was administered primarily through in-person, structured interviews at 3 and 6 months post-injury.^11^ Similar to the Citicoline Brain Injury Treatment (COBRIT) and the Progesterone for Traumatic Brain Injury Experimental Clinical Treatment (PROTECT III) studies, the TRACK-TBI Pilot administered the GOSE to capture disability related only to the TBI.^21,22^

### Statistical analysis

Descriptive statistics were reported using means and standard deviations (SDs) for continuous variables and proportions for categorical variables. The primary variable of interest was polytrauma, coded as present/absent. Univariate ordinal regressions were performed for the 3- and 6-month GOSE. Univariate logistic regressions were performed using clinical GOSE cutoffs for each time-point, as well as for the degree of change at 6 months compared with 3 months (improved, no change, or declined by GOSE). Multi-variable ordinal regressions were performed for the 3- and 6-month GOSE, controlling for known predictors from prior literature^23,24^ including age, sex, race, education, psychiatric history, high-speed mechanism of injury (motor vehicle accident [MVA] or pedestrian struck), GCS, and Marshall CT score (1: CT negative; 2: <5 mm shift, <25 mL lesion volume, cisterns present; 3–4: cisternal compression, >5 mm shift, no high/mixed-density lesion; and 5–6: surgical evacuation or high/mixed-density lesion >25 mL).^19^ GOSE 2–4 was combined into a single group to address small cell counts. Univariate and multi-variable odds ratios (ORs) and associated 95% confidence intervals (95% CIs) are reported for predictors. Statistical significance was assessed at *p* < 0.05. Analyses were performed using the Statistical Package for the Social Sciences (SPSS) version 25 (IBM Corporation, Chicago, IL).

## Results

Overall, 361 subjects met inclusion criteria. Table 1 presents overall demographics as well as differences by polytrauma status. Included subjects were aged 44.9 ± 18.9 years, 69.8% were male and 80.6% were Caucasian. Nearly 31% had a baseline psychiatric history and 13% had TBI by mechanism of assault. By GCS, 80.1% had mild TBI, 5.8% moderate TBI, and 14.1% severe TBI. Marshall CT score breakdown was Marshall score = 1: 48.2%, 2: 37.4%, 3–4: 8.0%, and 5–6: 6.4%. Polytrauma was present in 19.1%. The polytrauma group had more severe TBI (33.3 vs. 9.6%) and less mild TBI (60.9 vs. 84.6%), more high-speed injuries (MVA/pedestrian struck, 68.1 vs. 29.1%) and a higher proportion of Marshall score 2 injuries (47.8 vs. 34.9%), which were statistically significant.

**Table 1.** Demographics and Clinical Characteristics Compared across TBI+Polytrauma versus Isolated TBI Groups

	Total	TBI+Polytrauma		
Variable	(*n* = 361)	No (*n* = 292)	Yes (*n* = 69)	Sig. (*p*)
Age				0.337
Mean (SD)	44.9 (18.9)	45.3 (18.8)	42.9 (19.5)	
Sex				0.089
Male	252 (69.8%)	198 (67.8%)	54 (78.3%)	
Female	109 (30.2%)	94 (32.2%)	15 (21.7%)	
Race				0.572
Caucasian	291 (80.6%)	233 (79.8%)	58 (84.1%)	
African/AA	26 (7.2%)	23 (7.9%)	3 (4.3%)	
Other	44 (12.2%)	36 (12.3%)	8 (11.6%)	
Education				0.793
Mean (SD)	14.1 (2.9)	14.1 (2.9)	14.0 (2.8)	
Psychiatric history				0.242
No	251 (69.5%)	199 (68.2%)	52 (75.4%)	
Yes	110 (30.5%)	93 (31.8%)	17 (24.6%)	
Mechanism of injury				<0.001
Motor vehicle accident	86 (23.9%)	54 (18.6%)	32 (46.4%)^*^	
Pedestrian struck	46 (12.8%)	31 (10.7%)	15 (21.7%)^*^	
Fall from moving object	44 (12.2%)	40 (13.7%)	4 (5.8%)	
Fall from stationary	128 (35.6%)	113 (38.8%)	15 (21.7%)^*^	
Assault	47 (13.1%)	45 (15.5%)	2 (2.9%)^*^	
Other	9 (2.5%)	8 (2.7%)	1 (1.4%)	
Mechanism (high speed)				<0.001
No	229 (63.4%)	207 (70.9%)	22 (31.9%)	
Yes	132 (36.6%)	85 (29.1%)	47 (68.1%)	
Initial GCS				<0.001
3-8	51 (14.1%)	28 (9.6%)^*^	23 (33.3%)^*^	
9-12	21 (5.8%)	17 (5.8%)	4 (5.8%)	
13-15	289 (80.1%)	247 (84.6%)^*^	42 (60.9%)^*^	
Marshall CT score				0.016
1	174 (48.2%)	148 (50.7%)	26 (37.7%)	
2	135 (37.4%)	102 (34.9%)^*^	33 (47.8%)^*^	
3-4	29 (8.0%)	20 (6.8%)	9 (13.0%)	
5-6	23 (6.4%)	22 (7.5%)	1 (1.4%)	

High-speed mechanism = motor vehicle accident or pedestrian struck by vehicle.

AA, African-American; CT, computed tomography; GCS, Glasgow Coma Scale; SD, standard deviation; TBI, traumatic brain injury.

**FIG. 2.**
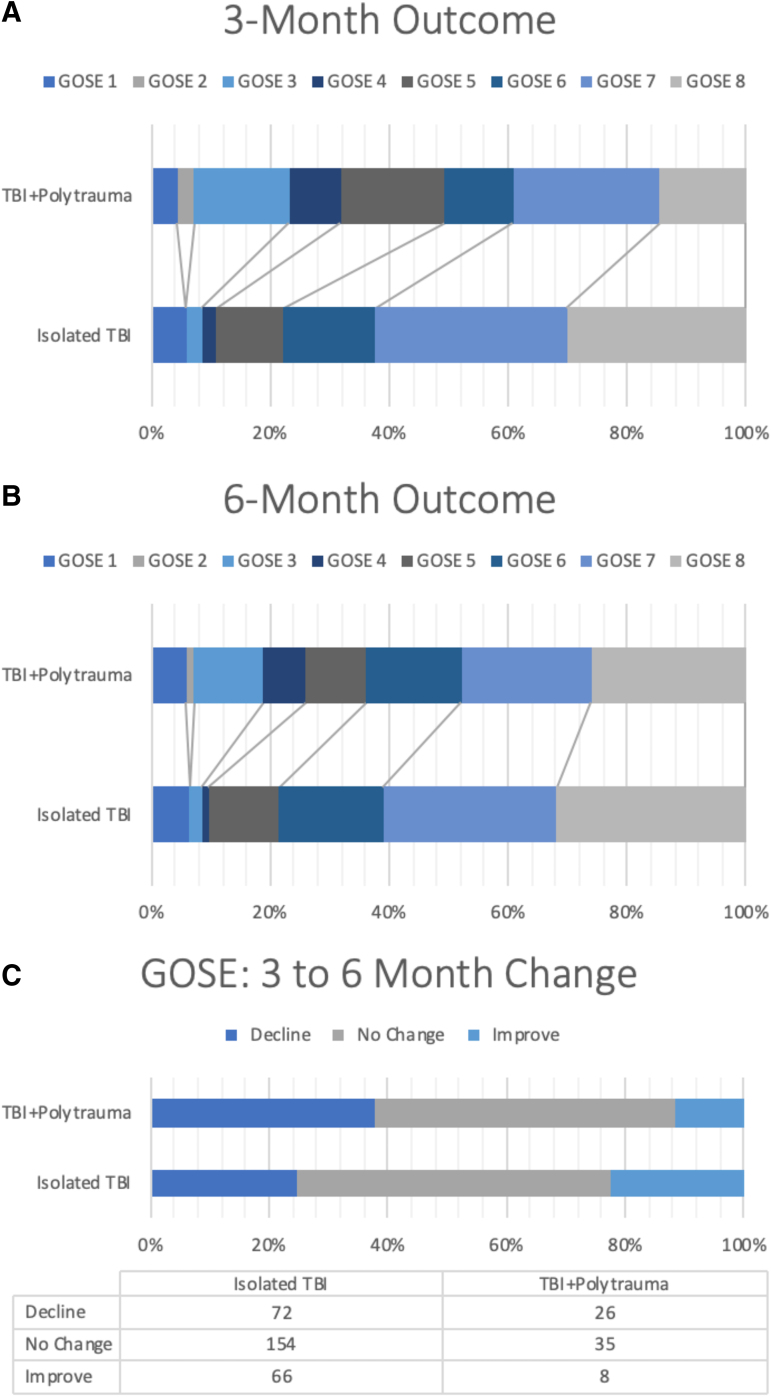
**(A,B)** Distribution of GOSE at 3 and 6 months between TBI patients with and without polytrauma. In general, the TBI+Polytrauma group had worse GOSE scores at 3 and 6 months compared with the Isolated TBI group. **(C)** Distribution of patients who declined, stayed the same, and improved in GOSE from 3 to 6 months. In the TBI+Polytrauma group, a statistically significantly greater proportion of patients declined and a smaller proportion of patients improved. GOSE, Glasgow Outcome Scale-Extended; TBI, traumatic brain injury.

At 3 months, the GOSE distribution consisted of expired (GOSE = 1, 5.5%), vegetative/severe disability (GOSE = 2–4, 9.4%), lower moderate disability (GOSE = 5, 12.5%), upper moderate disability (GOSE = 6, 14.7%), lower good recovery (GOSE = 7, 30.7%), and upper good recovery (GOSE = 8, 27.1%) (Fig. 2A). The 6-month GOSE distribution consisted of expired (6.4%), vegetative/severe disability (6.4%), lower moderate disability (11.6%), upper moderate disability (17.2%), lower good recovery (27.7%), and upper good recovery (30.7%) (Fig. 2B). Notably, compared with subjects without polytrauma, more subjects with polytrauma declined in GOSE from 3 to 6 months (37.7 vs. 24.7%), whereas fewer patients improved (11.6 vs. 22.6%, *p* = 0.033) (Fig. 2C).

**FIG. 3.**
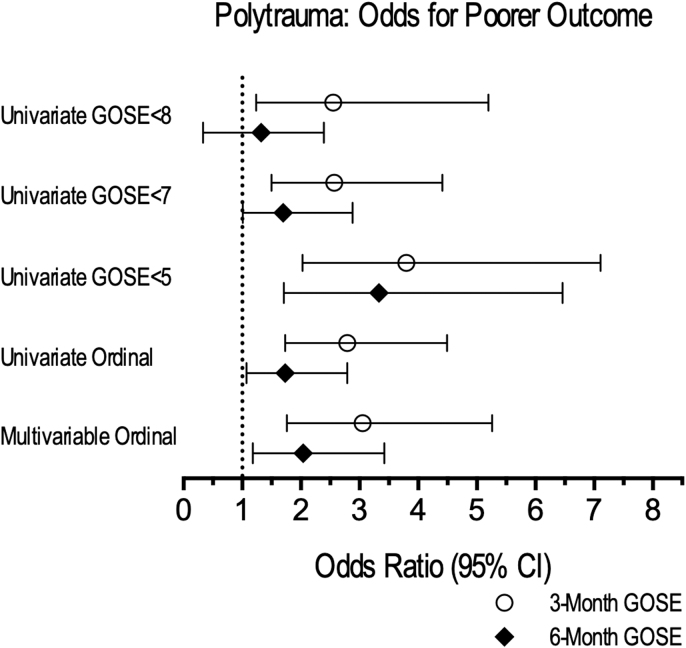
Polytrauma: univariate and multi-variable odds for poorer outcome. Odds ratios are shown for different known GOSE clinical cutoffs at 3 and 6 months for TBI+polytrauma (comparison group, with odds ratios shown) compared with Isolated TBI (reference group). Univariate logistic regressions are shown for GOSE <8 (any deficit vs. less than full recovery), GOSE <7 (moderate disability or worse vs. good recovery), and GOSE <5 (death/severe disability vs. moderate disability or better). Univariate ordinal regressions showed odds of worse outcome on the GOSE as an ordinal measure at 3 and 6 months. The multi-variable ordinal odds ratio controls are for known predictors of TBI outcome (age, sex, education, race, baseline psychiatric history, mechanism of injury, GCS score, and Marshall CT score). General trends were the same across all comparisons. Statistically significant odds ratios for worse outcome are associated with the TBI+Polytrauma group across all clinical cutoffs at 3 months, as well as for GOSE <7, GOSE <5, and univariate and multi-variable ordinal regressions. CT, computed tomography; GCS, Glasgow Coma Scale; GOSE, Glasgow Outcome Scale-Extended; TBI, traumatic brain injury.

On univariate ordinal regression, polytrauma was associated with greater odds for poorer GOSE at 3 months (OR = 2.79 [95% CI: 1.73-4.49]) and at 6 months (OR = 1.73 [95% CI: 1.07-2.79]) (Fig. 3). Univariate logistic regressions showed that polytrauma was associated with greater odds of “moderate disability or worse” (GOSE ≤6) at 3 months (OR = 2.57 [95% CI: 1.50-4.41]) and 6 months (OR = 1.70 [95% CI: 1.01-2.88]), as well as increased odds of “death/severe disability” (GOSE ≤4) at 3 months (OR = 3.80 [95% CI: 2.03-7.11]) and 6 months (OR = 3.33 [95% CI: 1.71-6.46]) (Fig. 2).

On multi-variable ordinal regression, the univariate relationships were conserved, and polytrauma was associated with poorer 3-month GOSE (OR = 3.05 [95% CI: 1.76-5.26]) and 6-month GOSE (OR = 2.04 [95% CI: 1.18-3.42]) (Fig. 2). Odds ratios for each predictor for 3-month and 6-month GOSE are shown in Supplementary Table S1.

## Discussion

In the prospective TRACK-TBI Pilot study, patients with TBI with concomitant polytrauma were at greater risk for worse disability at 3 and 6 months following injury than patients with isolated TBI. In addition, patients with TBI with polytrauma were more likely to decline, and less likely to improve, from 3 to 6 months after injury. Our data indicate that TBI with polytrauma constitutes a distinct cohort for prognostication and recovery. Patients with TBI with polytrauma may benefit from targeted follow-up care as well as improved outcome assessment tools to accurately capture disability attributable to TBI versus systemic injuries.

### Polytrauma confers greater risk for disability after TBI

The presence of polytrauma has been previously associated with more severe mechanisms of injury, higher initial injury severity score,^25^ and mortality.^26^ In our study, polytrauma was associated with a more severe GCS at presentation and higher Marshall scores, likely due to association with injuries of greater severity, higher force, and/or greater velocity. We observed a significantly higher incidence of polytrauma in MVA patients (68 vs. 29%), consistent with literature reporting an association of MVAs with polytrauma,^26,27^ higher injury severity scores,^27^ and less favorable GOSE compared with mechanisms with isolated TBI.^28^

It is not surprising that patients with polytrauma are at greater risk for poorer recovery, due to a combination of physiological and socioeconomic factors. Pathophysiologically, patients with polytrauma are at elevated risk for respiratory and multi-organ failure,^29,30^ coagulopathy,^5,31^ hypotension,^31^ systemic inflammation,^32^ and other predictors of mortality and poor outcome, in addition to delayed return to work^6^ and financial burden from hospitalization.^4^ Patients with polytrauma by definition have sustained additional injuries, any of which may impair long-term function,^33^ confer pain and disability,^34^ and therefore confound global measures of functional recovery such as the GOSE. Additionally, although we did not analyze the incidence of non-neurosurgical operative interventions, patients with polytrauma are more likely to require additional surgeries that in turn may prolong their hospitalization, rehabilitation, and recovery.^28^ All of this provides further impetus to ensure that these uniquely injured patients receive targeted, longitudinal, follow-up care that addresses both the brain injury and their peripheral injuries.

Our findings suggest a less favorable trajectory over time for patients who present with polytrauma, with worse initial GCS and worse GOSE (as indicated by both GOSE ≤4 and GOSE ≤6) at both 3 and 6 months. Of note, we observed a higher rate of decline in the GOSE from 3 to 6 months in patients with polytrauma. As such, not only do patients with polytrauma present with more severe injury, but they demonstrate impaired recovery and a comparatively increased risk of functional decline. The military literature has reported that patients with TBI, in particular those with polytrauma, often require comprehensive and lengthy physical rehabilitation.^35^

### Polytrauma confounds GOSE assessment as a TBI-specific measure

Our results reveal that the GOSE, as currently administered, can be confounded by polytrauma. The 8-point GOSE was designed to mitigate the limitations of the 5-point GOS, namely extracranial injuries and incorporation of more nuanced mental status changes.^7^ Initial validation studies were primarily in the moderate to severe TBI population,^36^ where TBI-related deficits likely superseded disability from other injuries.^7,37^ This is supported by the trauma literature, in which severity of cranial injury has been a consistent predictor of mortality.^29,38,39^ Whereas early studies document sufficiency of the GOSE in assessing subjective and/or neuropsychiatric symptoms across the spectrum of TBI,^40,41^ recent summative studies show ceiling effects for patients with mild TBI evaluated by GOSE.^42,43^ Specifically, the GOSE may lack items capable of objectively identifying and categorizing patients with milder impairment.^12^ This can further confound assessment of TBI outcome in the setting of polytrauma, given known risks of mortality and decreased work capacity associated with concomitant TBI reported in the trauma literature.^5,6,29^ Notably, all TRACK-TBI Pilot outcomes personnel were trained centrally to reference the GOSE to the TBI, which qualified the patient to enroll in the TRACK-TBI Pilot study.^11^

Consensus tools for evaluating outcome after TBI must be sensitive and specific in detecting attributable deficits from TBI. Our results show the critical need for an outcomes assessment tool that differentiates TBI-specific disability from systemic/overall disability, to have an accurate and precise end-point suitable for adoption across TBI care, research, and clinical trials. Understandably, the GOSE has remained in use in part due to its brevity, flexibility, reliability, and simplicity in administration^44^—important attributes to keep in mind for the development of future assessment tools.

### Insights and future direction

Historically, administration methods for the GOSE have varied. In general, U.S. drug trials (e.g., COBRIT,^21,45^ PROTECT III^22^), to the extent possible, have measured deficits attributable to brain injury, whereas European-based TBI multi-center studies (e.g., International Mission for Prognosis and Analysis of Clinical Trials in TBI [IMPACT]^46,47^ and Corticosteroid Randomization after Significant Head Injury [CRASH]^48,49^) have utilized the measure initially based on the 5-point GOS for outcome, and have employed the 8-point GOSE without patient- or clinician-based interpretation as to whether deficits are brain- or extracranial-specific.

We see in our results from the TRACK-TBI Pilot, which in the same manner as prior U.S. drug studies administered the GOSE to focus on brain-specific outcomes, that outcomes indeed differ for TBI with polytrauma compared with isolated TBI. Whether polytrauma is a confounder to brain-specific TBI outcome assessment, or whether it contributes to the extent of brain injury through a systemic mechanism (e.g., inflammation), is unknown. One way to study this would be to administer the GOSE with two queries for each question—the patient is asked to determine the perceived impact of disability attributable to their brain injury, and separately to their systemic/peripheral injuries, in the setting of polytrauma. In the ongoing multi-center Transforming Research and Clinical Knowledge in Traumatic Brain Injury study (http://tracktbi.ucsf.edu; NCT02119182), the standard GOSE assessment has been augmented to include such systematic documentation of peripheral injuries throughout the questionnaire. Peripheral injuries are listed in full, along with supplementary queries for each GOSE question regarding the perceived contribution of brain injury compared with peripheral injuries for each question on disability. This assessment tool is scored separately for overall and for TBI-specific disability. Results are currently under curation and analysis, and will be the subject of future publications.

The limitations of a single assessment tool for multi-dimensional TBI recovery is known,^42^ and current efforts should focus on further refinement of a concise battery of high-yield measures and/or a composite measure for TBI recovery. Until more sensitive measures are developed, the GOSE in its current iteration should be regarded as a disability measure to inform TBI outcome; however, caution should be employed when using it as a stand-alone measure in defining TBI outcome, especially in the setting of polytrauma.

### Limitations

We recognize several limitations in the analysis. TRACK-TBI Pilot enrolled patients under convenience sampling with associated biases and institution-specific patterns, affecting generalizability. Our study focused on patients who completed the GOSE at 3 and 6 months, hence patients who were lost to follow-up may constitute a different population with different risk factors and prognoses. Detailed surgical intervention data were not available for extracranial injuries, which may affect outcome. Likewise, granular data on inpatient medical management and referrals to outpatient services relating to polytrauma were not available. By study design, we were limited to the 3- and 6-month outcome time-points in accordance with the NINDS CDEs version 1,^8^ and did not have a more acute time-point during recovery, which may yield insights into acute and subacute trajectories. We utilized the Marshall CT score to classify and control for severity of intracranial lesions; however, CT-occult intracranial lesions such as axonal injury may confound evaluation and outcome.^50^ We focused on assessment of polytrauma using the GOSE as a global disability measure, and differences in more specific domains of functional and cognitive outcome between polytrauma and isolated TBI will be the topic of future investigations. Lastly, this is a study of association, and we cannot definitively make causal claims between risk factors and outcome in TBI with and without polytrauma.

## Conclusion

TBI patients with polytrauma are at greater risk for 3- and 6-month disability than patients with isolated TBI. Whether polytrauma is a confounder of brain-specific TBI outcome assessment because of the way that the GOSE is administered, or whether polytrauma contributes directly to the pathophysiology of TBI remains to be determined. Methodological improvements in assessing TBI-specific disability, versus disability attributable to systemic injuries, will generate better outcomes assessment tools in modern TBI care and research. The ongoing multi-center TRACK-TBI study administers the GOSE with two separate queries for each disability question to determine the perceived impact of disability attributable specifically to brain and systemic/peripheral injuries, with results pending.

## Supplementary Material

Supplemental data

## The TRACK-TBI Investigators

Shelly R. Cooper, Washington University in St. Louis, St. Louis, MO, USA; Kristen Dams-O'Connor, Icahn School of Medicine at Mount Sinai, New York, NY, USA); Wayne A. Gordon, Icahn School of Medicine at Mount Sinai, New York, NY, USA; Allison J. Hricik, University of Pittsburgh Medical Center, Pittsburgh, PA, USA; Andrew I. R. Maas, University Hospital Antwerp, Edegem, Belgium; David K. Menon, University of Cambridge, Cambridge, United Kingdom; Diane J. Morabito, University of California San Francisco, San Francisco, CA, USA.

## Notes

**Cite this article as:** Yue, JK, Satris, GG, Dalle Ore, CL, Huie JR, Deng, H, Winkler, EA, Lee, YM, Vassar, MJ., Taylor, SR, Schnyer, DM, Lingsma, HF, Puccio, AM, Yuh, EL, Mukherjee, P, Valadka, AB, Ferguson, AR, Markowitz, AJ, Okonkwo, DO, Manley, GT, and the TRACK-TBI Investigators (2020) Polytrauma is associated with increased 3- and 6-month disability after traumatic brain injury: A TRACK-TBI pilot study, *Neurotrauma Reports* 1:1, 32–41, DOI:10.1089/neur.2020.0004.

## Abbreviations Used

ADL: activity of daily living
AIS: Abbreviated Injury Score
CDEs: Common Data Elements
CI: confidence intervals
COBRIT: Citicoline Brain Injury Treatment
CRASH: Corticosteroid Randomization after Significant Head Injury
CT: computed tomography
DOD: Department of Defense
GCS: Glasgow Coma Scale
GOSE: Glasgow Outcome Scale-Extended
IMPACT: International Mission for Prognosis and Analysis of Clinical Trials in TBI
IRB: Institutional Review Board
MVA: motor vehicle accident
NINDS: National Institute of Neurological Disorders and Stroke
OR: odds ratio
PROTECT III: Progesterone for Traumatic Brain Injury Experimental Clinical Treatment
SD: standard deviation
SPSS: Statistical Package for the Social Sciences
TBI: traumatic brain injury
TRACK-TBI Pilot: Transforming Research and Clinical Knowledge in Traumatic Brain Injury Pilot

## References

[1] Maas, A.I.R., Menon, D.K., Adelson, P.D., Andelic, N., Bell, M.J., Belli, A., Bragge, P., Brazinova, A., Büki, A., Chesnut, R.M., Citerio, G., Coburn, M., Cooper, D.J., Crowder, A.T., Czeiter, E., Czosnyka, M., Diaz-Arrastia, R., Dreier, J.P., Duhaime, A.-C., Ercole, A., van Essen, T.A., Feigin, V.L., Gao, G., Giacino, J., Gonzalez-Lara, L.E., Gruen, R.L., Gupta, D., Hartings, J.A., Hill, S., Jiang, J.-Y., Ketharanathan, N., Kompanje, E.J.O., Lanyon, L., Laureys, S., Lecky, F., Levin, H., Lingsma, H.F., Maegele, M., Majdan, M., Manley, G., Marsteller, J., Mascia, L., McFadyen, C., Mondello, S., Newcombe, V., Palotie, A., Parizel, P.M., Peul, W., Piercy, J., Polinder, S., Puybasset, L., Rasmussen, T.E., Rossaint, R., Smielewski, P., Söderberg, J., Stanworth, S.J., Stein, M.B., von Steinbüchel, N., Stewart, W., Steyerberg, E.W., Stocchetti, N., Synnot, A., TeAo, B., Tenovuo, O., Theadom, A., Tibboel, D., Videtta, W., Wang, K.K.W., Williams, W.H., Wilson, L., Yaffe, K., and InTBIR Participants and Investigators. (2017). Traumatic brain injury: integrated approaches to improve prevention, clinical care, and research. Lancet Neurol. 16, 987–1048 29122524 10.1016/S1474-4422(17)30371-X

[2] Taylor, C.A., Bell, J.M., Breiding, M.J., and Xu, L. (2017). Traumatic brain injury-related emergency department visits, hospitalizations, and deaths - United States, 2007 and 2013. MMWR Surveill. Summ. 66, 1–16 10.15585/mmwr.ss6609a1 PMC5829835 28301451

[3] Xydakis, M.S., Ling, G.S.F., Mulligan, L.P., Olsen, C.H., and Dorlac, W.C. (2012). Epidemiologic aspects of traumatic brain injury in acute combat casualties at a major military medical center: a cohort study. Ann. Neurol. 72, 673–681 23060246 10.1002/ana.23757

[4] Yuan, Q., Liu, H., Wu, X., Sun, Y., Yao, H., Zhou, L., and Hu, J. (2012). Characteristics of acute treatment costs of traumatic brain injury in Eastern China–a multi-centre prospective observational study. Injury 43, 2094–2099 22595490 10.1016/j.injury.2012.03.028

[5] da Costa, L.G.V., Carmona, M.J.C., Malbouisson, L.M., Rizoli, S., Rocha-Filho, J.A., Cardoso, R.G., and Auler-Junior, J.O.C. (2017). Independent early predictors of mortality in polytrauma patients: a prospective, observational, longitudinal study. Clinics 72, 461–468 28954004 10.6061/clinics/2017(08)02 PMC5577616

[6] Gross, T., Schüepp, M., Attenberger, C., Pargger, H., and Amsler, F. (2012). Outcome in polytraumatized patients with and without brain injury. Acta Anaesthesiol. Scand. 56, 1163–1174 22735047 10.1111/j.1399-6576.2012.02724.x

[7] Wilson, J.T., Pettigrew, L.E., and Teasdale, G.M. (1998). Structured interviews for the Glasgow Outcome Scale and the extended Glasgow Outcome Scale: guidelines for their use. J. Neurotrauma 15, 573–585 9726257 10.1089/neu.1998.15.573

[8] Wilde, E.A., Whiteneck, G.G., Bogner, J., Bushnik, T., Cifu, D.X., Dikmen, S., French, L., Giacino, J.T., Hart, T., Malec, J.F., Millis, S.R., Novack, T.A., Sherer, M., Tulsky, D.S., Vanderploeg, R.D., and von Steinbuechel, N. (2010). Recommendations for the use of common outcome measures in traumatic brain injury research. Arch. Phys. Med. Rehabil. 91, 1650–1660.e17. 21044708 10.1016/j.apmr.2010.06.033

[9] Beers, S.R., Wisniewski, S.R., Garcia-Filion, P., Tian, Y., Hahner, T., Berger, R.P., Bell, M.J., and Adelson, P.D. (2012). Validity of a pediatric version of the Glasgow Outcome Scale-Extended. J. Neurotrauma 29, 1126–1139 22220819 10.1089/neu.2011.2272 PMC3325553

[10] Bagiella, E., Novack, T.A., Ansel, B., Diaz-Arrastia, R., Dikmen, S., Hart, T., and Temkin, N. (2010). Measuring outcome in traumatic brain injury treatment trials: recommendations from the traumatic brain injury clinical trials network. J. Head Trauma Rehabil. 25, 375–382 20216459 10.1097/HTR.0b013e3181d27fe3 PMC2939167

[11] Yue, J.K., Vassar, M.J., Lingsma, H.F., Cooper, S.R., Okonkwo, D.O., Valadka, A.B., Gordon, W.A., Maas, A.I.R., Mukherjee, P., Yuh, E.L., Puccio, A.M., Schnyer, D.M., Manley, G.T., and TRACK-TBI Investigators. (2013). Transforming research and clinical knowledge in traumatic brain injury pilot: multicenter implementation of the common data elements for traumatic brain injury. J. Neurotrauma 30, 1831–1844 23815563 10.1089/neu.2013.2970 PMC3814815

[12] Ranson, J., Magnus, B.E., Temkin, N., Dikmen, S., Giacino, J.T., Okonkwo, D.O., Valadka, A.B., Manley, G.T., Nelson, L.D., and TRACK-TBI Investigators. (2019). Diagnosing the GOSE: structural and psychometric properties using item response theory, a TRACK-TBI pilot study. J. Neurotrauma 36, 2493–2505 30907261 10.1089/neu.2018.5998 PMC6709724

[13] Yue, J.K., Winkler, E.A., Puffer, R.C., Deng, H., Phelps, R.R.L., Wagle, S., Morrissey, M.R., Rivera, E.J., Runyon, S.J., Vassar, M.J., Taylor, S.R., Cnossen, M.C., Lingsma, H.F., Yuh, E.L., Mukherjee, P., Schnyer, D.M., Puccio, A.M., Valadka, A.B., Okonkwo, D.O., Manley, G.T., and The Track-TBI Investigators. (2018). Temporal lobe contusions on computed tomography are associated with impaired 6-month functional recovery after mild traumatic brain injury: a TRACK-TBI study. Neurol. Res. 40, 972–981 30175944 10.1080/01616412.2018.1505416 PMC6464373

[14] Winkler, E.A., Yue, J.K., McAllister, T.W., Temkin, N.R., Oh, S.S., Burchard, E.G., Hu, D., Ferguson, A.R., Lingsma, H.F., Burke, J.F., Sorani, M.D., Rosand, J., Yuh, E.L., Barber, J., Tarapore, P.E., Gardner, R.C., Sharma, S., Satris, G.G., Eng, C., Puccio, A.M., Wang, K.K.W., Mukherjee, P., Valadka, A.B., Okonkwo, D.O., Diaz-Arrastia, R., Manley, G.T., and TRACK-TBI Investigators. (2016). COMT Val 158 Met polymorphism is associated with nonverbal cognition following mild traumatic brain injury. Neurogenetics 17, 31–41 26576546 10.1007/s10048-015-0467-8 PMC4988810

[15] Hildebrand, F., Giannoudis, P.V., van Griensven, M., Zelle, B., Ulmer, B., Krettek, C., Bellamy, M.C., and Pape, H.-C. (2005). Management of polytraumatized patients with associated blunt chest trauma: a comparison of two European countries. Injury 36, 293–302 15664594 10.1016/j.injury.2004.08.012

[16] Chen, C.-W., Chu, C.-M., Yu, W.-Y., Lou, Y.-T., and Lin, M.-R. (2011). Incidence rate and risk factors of missed injuries in major trauma patients. Accid. Anal. Prev. 43, 823–828 21376872 10.1016/j.aap.2010.11.001

[17] Maas, A.I., Harrison-Felix, C.L., Menon, D., Adelson, P.D., Balkin, T., Bullock, R., Engel, D.C., Gordon, W., Orman, J.L., Lew, H.L., Robertson, C., Temkin, N., Valadka, A., Verfaellie, M., Wainwright, M., Wright, D.W., and Schwab, K. (2010). Common data elements for traumatic brain injury: recommendations from the interagency working group on demographics and clinical assessment. Arch. Phys. Med. Rehabil. 91, 1641–1649 21044707 10.1016/j.apmr.2010.07.232

[18] Duhaime, A.-C., Gean, A.D., Haacke, E.M., Hicks, R., Wintermark, M., Mukherjee, P., Brody, D., Latour, L., Riedy, G., and Common Data Elements Neuroimaging Working Group Members, Pediatric Working Group Members. (2010). Common data elements in radiologic imaging of traumatic brain injury. Arch. Phys. Med. Rehabil. 91, 1661–1666 21044709 10.1016/j.apmr.2010.07.238

[19] Marshall, L.F., Marshall, S.B., Klauber, M.R., Van Berkum Clark, M., Eisenberg, H., Jane, J.A., Luerssen, T.G., Marmarou, A., and Foulkes, M.A. (1992). The diagnosis of head injury requires a classification based on computed axial tomography. J. Neurotrauma 9, Suppl. 1, S287–S292 1588618

[20] Vik, A., Nag, T., Fredriksli, O.A., Skandsen, T., Moen, K.G., Schirmer-Mikalsen, K., and Manley, G.T. (2008). Relationship of “dose” of intracranial hypertension to outcome in severe traumatic brain injury. J. Neurosurg. 109, 678–684 18826355 10.3171/JNS/2008/109/10/0678

[21] Zafonte, R., Friedewald, W.T., Lee, S.M., Levin, B., Diaz-Arrastia, R., Ansel, B., Eisenberg, H., Timmons, S.D., Temkin, N., Novack, T., Ricker, J., Merchant, R., and Jallo, J. (2009). The citicoline brain injury treatment (COBRIT) trial: design and methods. J. Neurotrauma 26, 2207–2216 19803786 10.1089/neu.2009.1015 PMC2824223

[22] Wright, D.W., Yeatts, S.D., Silbergleit, R., Palesch, Y.Y., Hertzberg, V.S., Frankel, M., Goldstein, F.C., Caveney, A.F., Howlett-Smith, H., Bengelink, E.M., Manley, G.T., Merck, L.H., Janis, L.S., Barsan, W.G., and NETT Investigators. (2014). Very early administration of progesterone for acute traumatic brain injury. N. Engl. J. Med. 371, 2457–2466 25493974 10.1056/NEJMoa1404304 PMC4303469

[23] Jacobs, B., Beems, T., Stulemeijer, M., van Vugt, A.B., van der Vliet, T.M., Borm, G.F., and Vos, P.E. (2010). Outcome prediction in mild traumatic brain injury: age and clinical variables are stronger predictors than CT abnormalities. J. Neurotrauma 27, 655–668 20035619 10.1089/neu.2009.1059

[24] Lingsma, H.F., Yue, J.K., Maas, A.I.R., Steyerberg, E.W., Manley, G.T., and TRACK-TBI Investigators. (2015). Outcome prediction after mild and complicated mild traumatic brain injury: external validation of existing models and identification of new predictors using the TRACK-TBI pilot study. J. Neurotrauma 32, 83–94 25025611 10.1089/neu.2014.3384 PMC4291219

[25] Cottington, E.M., Young, J.C., Shufflebarger, C.M., Kyes, F., Peterson, F.V. Jr, and Diamond, D.L. (1988). The utility of physiological status, injury site, and injury mechanism in identifying patients with major trauma. J. Trauma 28, 305–311 3351989 10.1097/00005373-198803000-00005

[26] El-Menyar, A., Consunji, R., Abdelrahman, H., Latifi, R., Wahlen, B.M., and Al-Thani, H. (2018). Predictors and time-based hospital mortality in patients with isolated and polytrauma brain injuries. World J. Surg. 42, 1346–1357 29063224 10.1007/s00268-017-4310-2

[27] Bushnik, T., Hanks, R.A., Kreutzer, J., and Rosenthal, M. (2003). Etiology of traumatic brain injury: characterization of differential outcomes up to 1 year postinjury. Arch. Phys. Med. Rehabil. 84, 255–262 12601658 10.1053/apmr.2003.50092

[28] Dagher, J.H., Habra, N., Lamoureux, J., De Guise, E., and Feyz, M. (2010). Global outcome in acute phase of treatment following moderate-to-severe traumatic brain injury from motor vehicle collisions vs. assaults. Brain Inj. 24, 1389–1398 20887096 10.3109/02699052.2010.523042

[29] El Mestoui, Z., Jalalzadeh, H., Giannakopoulos, G.F., and Zuidema, W.P. (2017). Incidence and etiology of mortality in polytrauma patients in a Dutch level I trauma center. Eur. J. Emerg. Med. 24, 49–54 26225615 10.1097/MEJ.0000000000000293

[30] Zhao, X.-J., Kong, L.-W., Du, D.-Y., and Su, H.-J. (2007). Analysis on care outcome of patients with polytrauma and coma. Chin. J. Traumatol. 10, 53–58 17229352

[31] Watanabe, T., Kawai, Y., Iwamura, A., Maegawa, N., Fukushima, H., and Okuchi, K. (2018). Outcomes after traumatic brain injury with concomitant severe extracranial injuries. Neurol. Med. Chir. 58, 393–399 10.2176/nmc.oa.2018-0116 PMC6156128 30101808

[32] Belavić, M., Jančić, E., Mišković, P., Brozović-Krijan, A., Bakota, B., and Žunić, J. (2015). Secondary stroke in patients with polytrauma and traumatic brain injury treated in an Intensive Care Unit, Karlovac General Hospital, Croatia. Injury 46, Suppl. 6, S31–S35 10.1016/j.injury.2015.10.057 26620118

[33] Mkandawire, N.C., Boot, D.A., Braithwaite, I.J., and Patterson, M. (2002). Musculoskeletal recovery 5 years after severe injury: long term problems are common. Injury 33, 111–115 11890911 10.1016/s0020-1383(01)00047-x

[34] van der Vliet, Q.M.J., Sweet, A.A.R., Bhashyam, A.R., Ferree, S., van Heijl, M., Houwert, R.M., Leenen, L.P.H., and Hietbrink, F. (2019). Polytrauma and high-energy injury mechanisms are associated with worse patient-reported outcomes after distal radius fractures. Clin. Orthop. Relat. Res. 477, 2267–2275 30985610 10.1097/CORR.0000000000000757 PMC6999931

[35] Swanson, T.M., Isaacson, B.M., Cyborski, C.M., French, L.M., Tsao, J.W., and Pasquina, P.F. (2017). Traumatic brain injury incidence, clinical overview, and policies in the U.S. Military health system since 2000. Public Health Rep. 132, 251–259 28135424 10.1177/0033354916687748 PMC5349478

[36] Teasdale, G.M., Pettigrew, L.E., Wilson, J.T., Murray, G., and Jennett, B. (1998). Analyzing outcome of treatment of severe head injury: a review and update on advancing the use of the Glasgow Outcome Scale. J. Neurotrauma 15, 587–597 9726258 10.1089/neu.1998.15.587

[37] Anderson, S.I., Housley, A.M., Jones, P.A., Slattery, J., and Miller, J.D. (1993). Glasgow Outcome Scale: an inter-rater reliability study. Brain Inj. 7, 309–317 8358404 10.3109/02699059309034957

[38] baum, j., entezami, p., shah, k., and medhkour, a. (2016). predictors of outcomes in traumatic brain injury. World Neurosurg. 90, 525–529 26721615 10.1016/j.wneu.2015.12.012

[39] Areas, F.Z., Schwarzbold, M.L., Diaz, A.P., Rodrigues, I.K., Sousa, D.S., Ferreira, C.L., Quevedo, J., Lin, K., Kupek, E., Ritter, C., Dal Pizzol, F., and Walz, R. (2019). Predictors of hospital mortality and the related burden of disease in severe traumatic brain injury: a prospective multicentric study in Brazil. Front. Neurol. 10, 432 31105642 10.3389/fneur.2019.00432 PMC6494964

[40] Wilson, J.T., Pettigrew, L.E., and Teasdale, G.M. (2000). Emotional and cognitive consequences of head injury in relation to the Glasgow Outcome Scale. J. Neurol. Neurosurg. Psychiatry 69, 204–209 10896694 10.1136/jnnp.69.2.204 PMC1737066

[41] Levin, H.S., Boake, C., Song, J., Mccauley, S., Contant, C., Diaz-Marchan, P., Brundage, S., Goodman, H., and Kotrla, K.J. (2001). Validity and sensitivity to change of the extended Glasgow Outcome Scale in mild to moderate traumatic brain injury. J. Neurotrauma 18, 575–584 11437080 10.1089/089771501750291819

[42] Nelson, L.D., Ranson, J., Ferguson, A.R., Giacino, J., Okonkwo, D.O., Valadka, A., Manley, G., and McCrea, M. (2017). Validating multidimensional outcome assessment using the TBI common data elements: an analysis of the TRACK-TBI pilot sample. J. Neurotrauma 34, 3158–3172 10.1089/neu.2017.5139 PMC5678361 28595478

[43] Silverberg, N.D., Crane, P.K., Dams-O'Connor, K., Holdnack, J., Ivins, B.J., Lange, R.T., Manley, G.T., McCrea, M., and Iverson, G.L. (2017). Developing a cognition endpoint for traumatic brain injury clinical trials. J. Neurotrauma 34, 363–371 27188248 10.1089/neu.2016.4443 PMC5220527

[44] McMillan, T., Wilson, L., Ponsford, J., Levin, H., Teasdale, G., and Bond, M. (2016). The Glasgow Outcome Scale—40 years of application and refinement. Nat. Rev. Neurol. 12, 477–485 27418377 10.1038/nrneurol.2016.89

[45] Zafonte, R.D., Bagiella, E., Ansel, B.M., Novack, T.A., Friedewald, W.T., Hesdorffer, D.C., Timmons, S.D., Jallo, J., Eisenberg, H., Hart, T., Ricker, J.H., Diaz-Arrastia, R., Merchant, R.E., Temkin, N.R., Melton, S., and Dikmen, S.S. (2012). Effect of citicoline on functional and cognitive status among patients with traumatic brain injury: Citicoline Brain Injury Treatment Trial (COBRIT). JAMA 308, 1993–2000 23168823 10.1001/jama.2012.13256

[46] Maas, A.I.R., Marmarou, A., Murray, G.D., Teasdale, S.G.M., and Steyerberg, E.W. (2007). Prognosis and clinical trial design in traumatic brain injury: the IMPACT study. J. Neurotrauma 24, 232–238 17375987 10.1089/neu.2006.0024

[47] Han, J., King, N.K.K., Neilson, S.J., Gandhi, M.P., and Ng, I. (2014). External validation of the CRASH and IMPACT prognostic models in severe traumatic brain injury. J. Neurotrauma 31, 1146–1152 24568201 10.1089/neu.2013.3003

[48] Roberts, I., and CRASH trial management group. (2002). The CRASH trial: the first large-scale randomized controlled trial in head injury. Corticosteroid Randomization After Significant Head injury. Natl. Med. J. India 15, 61–62 12044116

[49] van Leeuwen, N., Lingsma, H.F., Perel, P., Lecky, F., Roozenbeek, B., Lu, J., Shakur, H., Weir, J., Steyerberg, E.W., Maas, A.I.R., International Mission on Prognosis and Clinical Trial Design in TBI Study Group, Corticosteroid Randomization After Significant Head Injury Trial Collaborators, and Trauma Audit and Research Network. (2012). Prognostic value of major extracranial injury in traumatic brain injury: an individual patient data meta-analysis in 39,274 patients. Neurosurgery 70, 811–8; discussion 818. 21904253 10.1227/NEU.0b013e318235d640

[50] Yuh, E.L., Mukherjee, P., Lingsma, H.F., Yue, J.K., Ferguson, A.R., Gordon, W.A., Valadka, A.B., Schnyer, D.M., Okonkwo, D.O., Maas, A.I.R., Manley, G.T., and TRACK-TBI Investigators. (2013). Magnetic resonance imaging improves 3-month outcome prediction in mild traumatic brain injury. Ann. Neurol. 73, 224–235 23224915 10.1002/ana.23783 PMC4060890

