# Supplementary material for: Polytrauma Is Associated with Increased Three- and Six-Month Disability after Traumatic Brain Injury: A TRACK-TBI Pilot Study
Source: Neurotrauma Rep. 2020 Jul 23;1(1):32–41. doi: 10.1089/neur.2020.0004 (PMC8240880; doi:10.1089/neur.2020.0004)
Supplement: Supplemental data [file Supp_Table1.pdf]

Supplementary Data

Supplementary Table S1. Multinomial Ordinal Regression for 3-Month and 6-Month GOSE

	3-Month GOSE			*6-Month GOSE*		
Predictor	***Odds Ratio***	***95% CI***	***Sig. (p)***	***Odds Ratio***	***95% CI***	***Sig. (p)***
Cohort						
Isolated TBI	Reference	—	—	Reference	—	—
TBI+Polytrauma	3.05	1.76-5.26	<0.001	2.04	1.18-3.42	0.010
Age						
Per-year change	1.02	1.01-1.03	<0.001	1.02	1.01-1.03	<0.001
Sex						
Male	Reference	—	—	Reference	—	—
Female	1.14	0.74-1.74	0.561	1.16	0.76-1.78	0.489
Race						
Caucasian	Reference	—	—	Reference	—	—
African/AA	2.51	1.24-5.08	0.010	1.27	0.62-2.62	0.510
Other	1.32	0.73-2.39	0.366	1.36	0.76-2.45	0.300
Education						
Per-year change	0.93	0.86-0.99	0.024	0.88	0.82-0.95	<0.001
Psychiatric history						
No	Reference	—	—	Reference	—	—
Yes	1.97	1.28-3.03	0.002	2.00	1.30-3.08	0.002
Mechanism (high speed)						
No	Reference	—	—	Reference	—	—
Yes	0.75	0.49-1.16	0.201	0.62	0.40-0.95	0.029
Initial GCS						
13-15	Reference	—	—	Reference	—	—
3-12	3.96	2.16-7.25	<0.001	3.53	1.97-6.36	<0.001
Marshall CT score						
1	Reference	—	—	Reference	—	—
2	1.94	1.23-3.06	0.005	0.94	0.59-1.48	0.780
3-4	5.65	2.64-12.08	<0.001	3.00	1.40-6.39	0.005
5-6	14.74	5.52-39.37	<0.001	6.92	2.66-18.00	<0.001

Multi-variable ordinal regression for TBI+Polytrauma versus Isolated TBI with odds ratios and significance values displayed for variables in the model for worse ordinal outcome on GOSE. Variables are known predictors for TBI outcome. After controlling for covariates, TBI+Polytrauma was associated with increased odds ratios for poorer ordinal outcome on 3- and 6-month GOSE.

AA, African-American; CI, confidence interval; CT, computed tomography; GCS, Glasgow Coma Scale; GOSE, Glasgow Outcome Scale-Extended; TBI, traumatic brain injury.
